# The Effects of the Lactation Period, Mare Age, and Foaling on the Chemical and Physical Composition of Milk from Kazakh Mares Kept Under Natural Pasture Conditions

**DOI:** 10.3390/ani15121817

**Published:** 2025-06-19

**Authors:** Maxat Toishimanov, Olzhas Zhanten, Rakhim Kanat, Indira Beishova, Vadim Ulyanov, Tolegen Assanbayev, Tlekbol Sharapatov, Dias Daurov, Ainash Daurova, Zagipa Sapakhova, Askar Nametov, Malika Shamekova

**Affiliations:** 1 Laboratory of Breeding and Biotechnology, Institute of Plant Biology and Biotechnology, Timiryazev 45, Almaty 050040, Kazakhstan; m.toishmanov@ipbb.kz (M.T.); o.zhanten@ipbb.kz (O.Z.); r.kanat@ipbb.kz (R.K.);; 2 Tanir Research Laboratory, Al-Farabi Avenue 75B, Almaty 050060, Kazakhstan; 3 Testing Center, Zhangir Khan West-Kazakhstan Agrarian Technical University, Zhangir Khan 51, Oral 090009, Kazakhstan; indirabeishova85@gmail.com (I.B.); vadimkst19@gmail.com (V.U.); anametov@gmail.com (A.N.); 4 Zootechnology and Veterinary Medicine, Faculty of Agriculture Science, Toraighyrov University, Lomov 64, Pavlodar 140008, Kazakhstan; asanbaev.tolegen.shonaevich@teachers.tou.edu.kz (T.A.); sharapatov.ts@teachers.tou.edu.kz (T.S.)

**Keywords:** Kazakh mare, lactation period, foaling, milk composition, physical properties, dairy quality, equine milk production

## Abstract

This study aimed to evaluate the chemical composition of milk from Kazakh mares kept under free-grazing conditions on natural pasture without supplementary feeding. Fifty Kazakh mares were aged 3 to 13 years, kept in herds, and categorized by age, number of foalings, and lactation stage for statistical analysis. Milk samples were collected, chilled, and analyzed on the same day using a Milkoscan FT2. The composition of fat, protein, lactose, total solids, casein, and other components was examined. Statistical analyses, including ANOVA, PCA, CDA, and Pearson correlation analysis, were performed using JMP 17 Pro to assess the effects of lactation stage, mare age, and foaling number and to identify key relationships. The results showed that milk composition was significantly influenced by these factors. Milk from the late-lactation stage contained higher protein, lactose, and total nutrients, while milk from the early-lactation stage had a higher sugar content and acidity. Older mares and those with higher foalings produced milk with higher concentrations of glucose and galactose, while younger mares and those with fewer foalings produced milk with higher fat. A key aspect of this experiment was that all mares grazed exclusively on natural pastures without supplementation, allowing for an objective assessment of their inherent productive potential. These findings deepen the understanding of mare milk composition dynamics in Kazakh breed horses under extensive management, supporting its potential for organic dairy production.

## Simple Summary

Mare milk is a traditional and valuable product in Kazakhstan, especially in rural and nomadic communities. In this study, we examined the quality of milk from mares kept exclusively on natural pastures, without any supplementary feeding. This made it possible to objectively assess the natural qualities of their milk. We found that milk produced in late lactation had more protein, lactose, and total nutrients, while milk produced in early lactation had higher sugar and acidity. Older mares and those with the highest foalings produced milk with higher concentrations of glucose and galactose, while younger mares and those with fewer foalings produced milk with higher fat. Our findings suggest that mares aged 4–5 years with 1–2 foals may produce the best nutrient balance in milk. Overall, the mares of the Kazakh breed demonstrated consistently high values for lactose, milk density, total solids, protein, and casein despite the lack of supplemental feeding and their exclusive pasture-based diet. These results highlight the value of the indigenous Kazakh horse as a genetic resource for dairy horse breeding, including for organic production, due to the absence of processed and concentrated feeds in their diet.

## 1. Introduction

The Kazakh horse, a breed indigenous to Central Asia, is known for its resilience, endurance, and adaptability to the harsh conditions of the region. The mares of this breed play a crucial role in the sustenance of the livestock and agricultural practices in the area, especially in pastoral communities, where the horses are kept for both riding and milk production. Raw milk from Kazakh mares is traditionally used to produce kumis. Mare milk is a dairy product highly valued for its nutritional content and medicinal properties in Kazakh culture [1,2].

Kazakh mares are medium-sized, with a strong, compact build that allows them to thrive in the harsh, variable climate of Kazakhstan, where temperatures can fluctuate drastically between seasons. Their coat is typically thick and weather-resistant, which helps them cope with both the extreme cold in winter and the intense heat in summer. This breed is known for its stamina and ability to travel long distances, making it highly valuable in pastoral economies where movement between grazing lands is common. Kazakh horses have also been bred for their hardiness in pasture-based systems. They graze on a variety of natural forage found in the vast steppe regions, and their ability to thrive on diverse, often sparse, vegetation makes them well suited to the extensive pastoral practices of the region. These horses are also utilized for their meat and skins, further adding to their value in the local economy [3,4,5].

Over the years, mares of the Kazakh breed have maintained a strong genetic lineage with minimal crossbreeding, which contributes to their resilience and unique characteristics [6,7,8]. Pasture conditions play a critical role in lactation in mares, particularly in regions such as Kazakhstan, where horses are raised primarily in extensive systems, grazing on natural pastures [9]. The nutritional quality and availability of pastures directly influence the quantity and composition of the milk produced by mares during the lactation period [10,11]. As mares rely on the nutrients available in their grazing environment, variations in pasture conditions—such as forage type, soil quality, climate, and seasonal fluctuations—can significantly impact their overall health and milk production. Pasture-based systems are inherently dependent on the natural environment, and these systems are characterized by the variability of forage quality throughout the year. During the growing season, when pastures are lush and nutrient-dense, mares typically have access to high-quality forage that can support optimal milk production. However, in periods of drought, overgrazing, or winter when pasture growth slows, the quality and availability of forage can decrease, which may negatively affect milk yield and nutritional composition [12]. Moreover, seasonal changes are another factor influencing pasture conditions and, by extension, lactation. For instance, during early spring, pastures may provide a surge of fresh, high-quality forage, contributing to increased milk production. As the seasons change and pastures mature, the nutritional content can fluctuate, impacting the mare’s energy levels and milk composition. In regions with extreme climates, such as Kazakhstan, mares may face nutritional challenges during the winter months when pasture access is limited, potentially leading to reduced lactation performance [13].

Other horse breeds in Central Asia and neighboring regions share similar adaptive traits. The Altai horse, native to southern Siberia and western Mongolia, is a robust, small-framed breed well known for its high-fat milk and tolerance to mountainous terrain and harsh winters [14]. The Mongolian horse, an ancient landrace central to nomadic life in Mongolia, is hardy and disease-resistant, and it provides milk widely used for fermented products such as airag (traditional Mongolian kumis) [15,16]. The Yanqi horse of Xinjiang, China, is a local Chinese breed valued for both riding and milk production, and studies have shown that it produces milk with relatively high protein and lactose contents, making it favorable for processing and fermentation [17,18]. The Russian heavy draft (Russkaya Tyazhelovoz) is not only a powerful workhorse but has also been reported to produce milk with higher fat and total solid contents, making it suitable for dairy use in rural regions [19]. The Vyatka horse, a rare and resilient breed from the Ural region, is adapted to severe winter climates and performs well on sparse forage, showing promise for small-scale milk production systems [20]. The Don horse, originally bred in southern Russia, is a light saddle breed valued for its endurance and traditional role in military and agricultural life; in some regions, Don mares are also milked for traditional fermented beverages [21]. These Russian breeds, similar to their Kazakh and Mongolian counterparts, exemplify the diversity of equine dairy potential in the Eurasian steppe and forest–steppe zones [22].

The composition of mare milk varies throughout the lactation period, influenced by factors such as the lactation stage, foal age, and environmental conditions. Understanding the physical composition of mare milk is essential for improving the productivity of Kazakh horses in pasture-based systems and ensuring the health and wellbeing of both mares and foals. Pasture conditions, with their natural variability in nutrition and climate, further contribute to the fluctuating quality of milk produced by mares [23,24,25,26,27].

The physical composition of milk, including its fat, protein, lactose, and mineral contents, undergoes significant changes from the early to late stages of lactation and based on the mare’s reproductive history and age. These changes, in turn, affect foal development and the nutritional value of the milk [28].

In this context, this study aimed to examine the effect of the lactation period, foaling, and mare age on the physical composition of milk obtained from Kazakh breed mares kept under natural pasture conditions. By analyzing how milk composition varies with lactation stages, mare age, and foaling number, this research provides valuable insights to inform improved management practices for Kazakh horses and enhance the quality of mare milk for both human consumption and foal nutrition.

The aim of this study was to address key questions regarding how pasture conditions, lactation timing, and foaling impact the physical properties of mare milk, ultimately enhancing the productivity and sustainability of horse husbandry in Central Asia.

## 2. Materials and Methods

### 2.1. Study Design and Sample Collection

This study aimed to assess the chemical composition of milk obtained from Kazakh mares kept under free-grazing conditions on natural pasture, without any supplementary feeding. For this purpose, a farm was found where horses are kept only in herds with no additional feeding (only natural pastures) and where the milking factor has a minimal effect on foals.

The study involved 50 Kazakh mares kept under natural pasture conditions at the “BIRLIK” farming enterprise located in Akkuli District of Pavlodar Region (northeastern Kazakhstan). The animals were maintained without any supplementary feeding. All mares foaled in April. The mares were breed-typical, uniform in type, and in good condition, while the foals exhibited well-proportioned physical development and good body condition.

The age of the mares included in this study ranged from 3 to 13 years. The samples were divided into groups based on age—first-foaling mares, 3 years old (*n* = 9); young mares, 4–5 years old (*n* = 21); and mature mares, over 6 years old (*n* = 20)—and based on the foaling number—one foaling (*n* = 12), two foalings (*n* = 12), three foalings (*n* = 6), and four or more foalings (*n* = 20).

The lactation period of the studied mares lasted for six months, from April to September. Although the mares were milked daily from April to September, milk samples for this study were collected only twice per mare—once in the third month (early lactation) and once in the fifth month (late lactation)—for compositional analysis. The mares were milked twice a day from 9:00 to 13:00 and from 15:00 to 19:00, from May to September. During the first month of lactation, the mares were not milked to allow the foals full access to the milk.

Each mare was pre-numbered for individual identification during the experiment. The mares were herded to the milking area at around 7:00 a.m., and the foals were temporarily separated from them during milking using a partition. A special foal was used to stimulate milk letdown in mares that delayed ejection; the foal was briefly allowed to suckle until milk flow began, at which point it was removed, and the milking machine was attached. This is a traditional technique used in the milking of herd-managed mares, distinct from the practices used in intensive dairy horse production. Milk samples were collected in the first half of the day. Before each milk collection, the teat area was cleaned with fresh water and thoroughly dried. Milking was performed using a portable Melasty-brand milking machine originally designed for goats. As horse-specific milking equipment is not commercially standardized, the machine was adapted for mare use by adjusting the vacuum pressure and pulsation rate to simulate the natural suckling behavior of foals. Soft silicone teat liners were used, and the suction parameters were carefully calibrated to suit equine teat anatomy and avoid discomfort. This approach allowed for efficient and safe milk extraction under pasture-based conditions and reflects a traditional but practically modified method for milking herd-managed mares. Using a milking machine, each mare was milked into separate buckets. The milk in the bucket was thoroughly mixed, and a 50 mL sample was placed into a sterile polypropylene tube. The milk samples were immediately placed in a portable refrigerated container and transported to the laboratory on the same day for analysis.

The chemical compositions of the collected samples were analyzed on the same day.

### 2.2. Pasture Characteristics

For this experiment, we selected a farm that did not provide the horses with any supplemental feed. Their diet consisted exclusively of natural steppe pastures typical of the region. These pastures were dominated by feather–fescue, feather grass–wormwood, and feather grass–wormwood–fescue plant communities growing on sandy and sandy loam soils. The herbaceous composition of the grazing areas included *Stipa lessingiana*, *Stipa capillata*, *Festuca sulcata*, *Artemisia marschalliana*, *Artemisia campestris*, *Artemisia austriaca*, and *Potentilla acaulis*. These pastures provide seasonal variation in nutrient availability, reflecting the natural foraging conditions of the region [29].

### 2.3. Quality Composition Determination of Mare Milk

To examine the chemical composition of the milk, the fat, protein, solids-not-fat (SNF), total solids (TS), lactose, galactose, glucose, freezing point, acidity, lactic acid, density, citric acid, urea, and casein were measured using an automated infrared analysis with a Milkoscan FT2 instrument (Foss Electric, Hillerod, Denmark). As standard factory calibrations are typically optimized for bovine milk, a customized calibration curve was developed using reference samples of mare milk with known composition, previously analyzed using classical chemical methods (e.g., Kjeldahl for protein and Gerber for fat). Calibration accuracy was verified by comparing the predicted values with the laboratory reference data, ensuring a reliable quantification of milk components specific to mares of the Kazakh breed.

### 2.4. Statistical Analysis

All data were analyzed using JMP 17 Pro (JMP, Cary, NC, USA). Descriptive statistics were computed for all milk composition variables to summarize the means and standard deviations across groups. A general linear model of separate one-way analysis of variance (ANOVA) was applied with three fixed factors: lactation period (early vs. late), mare age group (3 years, 4–5 years, and ≥6 years), and foaling number (1, 2, 3, and ≥4) on the physical and chemical parameters of the mare milk. Each factor was analyzed independently. The significance level was set to *p* < 0.05, and Tukey’s HSD post hoc test was used for multiple comparisons where applicable.

To explore multivariate relationships and identify patterns in the milk compositions across groups, a principal component analysis (PCA) was conducted using standardized data. The PCA was used to visualize the associations among milk traits and their relationship with mare age, lactation stage, and foaling number.

In addition, a canonical discriminant analysis (CDA) was employed to determine whether lactation period, mare age, or foaling number could significantly discriminate between groups based on the full set of milk composition variables. The canonical structure matrix and eigenvalues were used to interpret the relative contribution of the variables to group separation. The statistical significance of the canonical functions was tested using Wilks’ lambda with F-approximation.

Pearson correlation coefficients were calculated between all milk composition parameters to assess the strength and direction of linear relationships. The results were visualized in a correlation matrix heatmap.

## 3. Results

### 3.1. Effects of Lactation Stage, Mare Age, and Foaling Number on Milk Composition

Table 1 presents the mean values of the milk composition parameters by lactation stage, age group, and foaling number. Overall, significant differences were observed across the groups. Mare milk quality variations were confirmed using Tukey’s HSD test (*p* < 0.05).

The protein content increased significantly in the late-lactation milk (1.958 ± 0.091%) compared to in the early-lactation milk (1.819 ± 0.032%), as confirmed using Tukey’s HSD post hoc test (*p* = 0.0003). Similarly, SNF and TS were significantly higher in the late-lactation milk (9.888 ± 0.206% and 10.363 ± 0.322%) than in the early-lactation milk (9.354 ± 0.487% and 9.813 ± 0.623%) (*p* = 0.0001 for both). The lactose concentration increased from 6.741 ± 0.307% in the early-lactation milk to 7.073 ± 0.202% in the late-lactation milk (*p* = 0.0001). Additionally, the freezing point significantly decreased in the late-lactation milk (−0.588 ± 0.014 °C) compared to in the early-lactation milk (−0.528 ± 0.034 °C), indicating a higher content of dissolved solids (*p* = 0.0001). Mares aged 4–5 years produced milk with the highest protein (1.917 ± 0.021%), SNF (9.682 ± 0.410%), and TS (10.163 ± 0.473%) levels, although these differences were not statistically significant according to Tukey’s test (*p* > 0.05). The fat content was significantly higher in the milk from younger mares (3 years, 0.421 ± 0.044%) than in the milk from older mares (≥6 years, 0.351 ± 0.022%) (*p* = 0.0194), with the post hoc analysis confirming the difference. The glucose and galactose concentrations were significantly higher in the milk from mares aged 3 years old (0.266 ± 0.064% and 0.338 ± 0.049%, respectively) compared to those from mares aged 4–5 but were not significantly different from the group aged 6+ years, supported by post hoc significance (*p* = 0.0038 and *p* = 0.0438). The milk from mares aged 6 years or above showed significantly lower urea (23.523 ± 0.810 mg/dL) and density (1040.520 ± 3.801 g/L) values than the milk from younger mares, as confirmed with Tukey’s test (*p* < 0.05). In contrast, the acidity (7.978 ± 0.142 °D) and lactic acid (0.099 ± 0.001%) were significantly lower in the milk from older mares, suggesting reduced fermentative activity (*p* = 0.0088 and *p* = 0.0071, respectively). The milk freezing point was the lowest in the 4–5-year-old group (−0.570 ± 0.043 °C), significantly differing from that in both the younger and older age groups (*p* = 0.0042), indicating increased levels of dissolved solids.

The fat content decreased significantly with an increasing foaling number, with the milk from mares with three foalings showing the lowest fat level (0.300 ± 0.052%) compared to the milk from mares with one or two foalings (0.441 ± 0.038% and 0.418 ± 0.037%, respectively), confirmed using Tukey’s HSD test (*p* = 0.0129). The freezing point was also significantly affected by foaling number, with the lowest value observed in the milk from mares with three foalings (−0.583 ± 0.034 °C), differing significantly from that in the other groups (*p* = 0.0023). The acidity and lactic acid levels were significantly lower in the milk from mares with four or more foalings (7.978 ± 0.142 °D and 0.099 ± 0.001%, respectively) than in the milk from mares with fewer foalings (*p* = 0.0152 and *p* = 0.0126). The milk density varied significantly among groups, with the lowest value also observed for mares with ≥4 foalings (1040.520 ± 3.801 g/L) (*p* = 0.0196). The urea content remained stable in milk from mares with one to three foalings but was significantly lower in milk from mares with four or more foalings (23.523 ± 0.810 mg/dL), indicating a potential decline in milk nitrogen content associated with repeated foaling (*p* = 0.0015), confirming an effect of reproductive history on milk nitrogen content.

### 3.2. Principal Component Analysis

In the PCA, variables such as lactose, SNF, total solids, protein, casein, density, and citric acid were positively associated with PC1 and aligned with the samples obtained during late lactation. In contrast, the freezing point was positioned negatively along both PC1 and PC2 and was associated with the samples obtained during early lactation. Other variables, including fat, urea, acidity, glucose, and galactose, showed no clear group-specific associations and were broadly distributed, especially across mare age and foaling categories. As a result, clear separation was observed only by lactation stage.

Regarding age and foaling effects, older mares and those with more foalings produced milk richer in fat, urea, and acidity, similar to early-lactation milk. Younger mares and those with fewer foalings produced milk with higher glucose and galactose levels, which are also characteristic of early-lactation milk. Protein, TS, SNF, and lactose peaked in the milk from mid-aged mares (4–5 years), mares with 1–2 foalings, and late lactation, indicating that these groups may be optimal for dairy production. The freezing point reduction pattern matched between milk from early lactation and younger mares, suggesting a general trend of higher dissolved solids in milk from freshly lactating mares.

**Figure 1:**
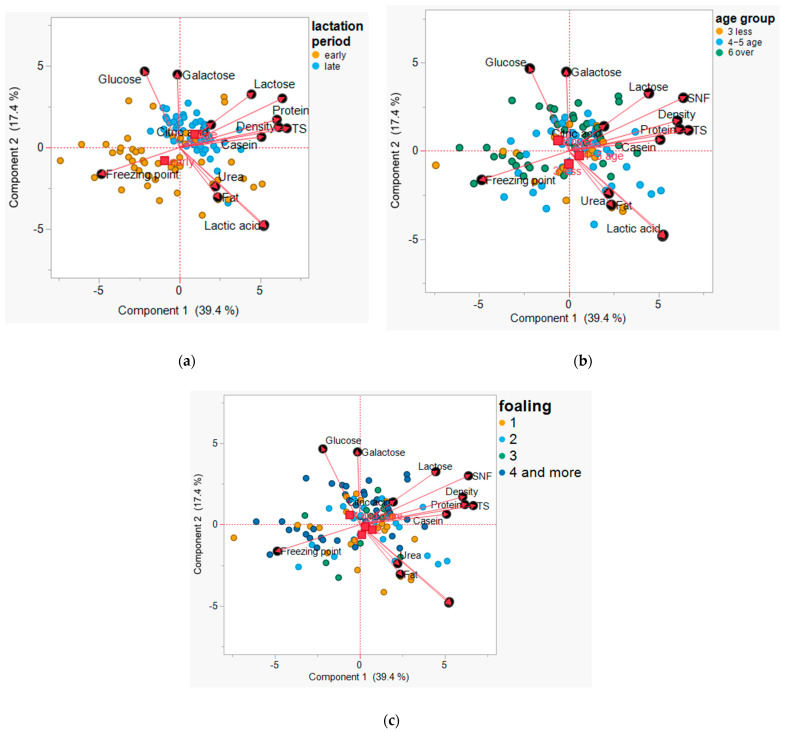
Principal component analysis biplots of mare milk composition by (**a**) lactation period, (**b**) mare age, and (**c**) foaling number.

As shown in Figure 1, the PCA biplot illustrates the relationships between the different milk composition parameters and the distribution of mare age groups (3 years, 4–5 years, and 6 years or over). The first principal component (PC1) explains 39.4% of the total variance, while the second principal component (PC2) explains 17.4%. Together, these components account for 56.8% of the total variance in the dataset. Glucose and galactose are positively associated with PC2; however, no distinct separation is observed for mares aged 3 years in the PCA plot. While lactose, SNF, density, protein, and casein contribute positively to PC1, samples from different mare age groups, including 4–5 years, appear broadly distributed along this axis, indicating no clear age-related separation. Fat, urea, acidity, and lactic acid are negatively correlated with PC2; however, samples from mares older than 6 years are broadly distributed. The freezing point appears to be negatively associated with PC1 and PC2, positioned away from most other parameters. The 4–5-year-old group is centered around casein, protein, and density, indicating that milk from this group is richer in protein content. Mares 6 years or older have strong associations with fat, urea, and acidity, suggesting significant metabolic shifts affecting these components. Younger mares produce milk richer in sugars (glucose and galactose), while older mares produce milk with higher levels of fat, urea, and acidity. The 4–5-year-old mares appear to produce milk with a balanced composition, featuring higher protein and casein contents.

Glucose and galactose are positively associated with PC2, meaning that they are more prominent in the milk from mares with fewer foalings. Lactose, SNF, density, TS, and protein show a strong positive correlation with PC1, indicating that the milk from mares with fewer foalings tends to have higher values of these parameters. Fat, urea, and acidity are positioned negatively along PC1, suggesting that the milk from mares with more foalings (four or more) has higher concentrations of these components. The freezing point is negatively correlated with PC1 and PC2, appearing to be more associated with the milk from mares with fewer foalings. Mares with one and two foalings are more associated with higher lactose, SNF, protein, and TS, which indicates that their milk composition is richer in total solids and proteins. The milk from mares with three and four or more foalings exhibits higher fat, urea, and acidity, suggesting that repeated foaling leads to metabolic changes affecting milk composition. The freezing point is lower in the milk from mares with fewer foalings, which may be linked to its higher concentration of dissolved solids.

### 3.3. Correlation Analysis

**Figure 2:**
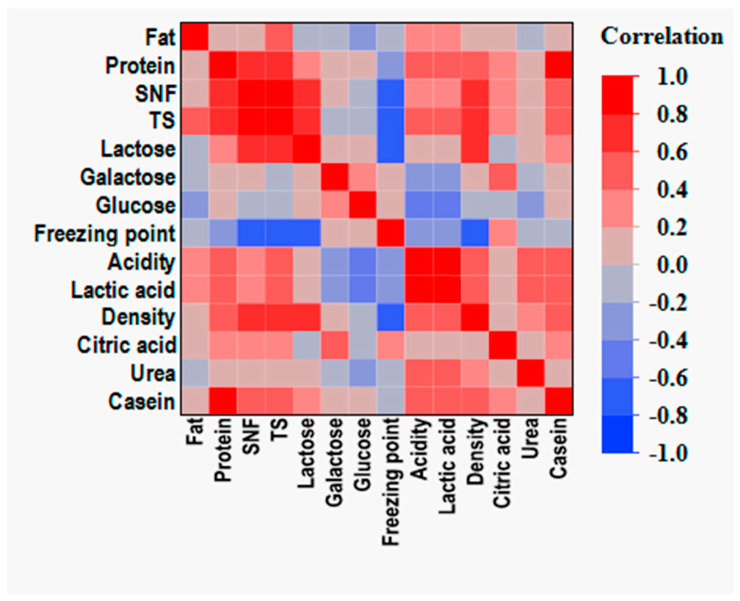
Correlation matrix of mare milk composition parameters.

Protein, SNF, TS, and lactose show a strong positive correlation with each other, indicating that a higher protein content is associated with increased total solids and lactose levels, as shown in Figure 2. Casein and protein have a strong correlation, suggesting that casein content is a significant contributor to total protein levels in mare milk. Density and SNF are highly correlated, which implies that the solids-not-fat fraction significantly influences milk density. Lactic acid and acidity show a positive correlation, indicating that lactic acid is a likely contributor to the overall acidity of mare milk. In contrast, citric acid exhibits only a weak positive correlation with acidity, suggesting that it plays a minor role in determining the acid profile under the studied conditions.

The freezing point and SNF show a strong negative correlation, indicating that a higher SNF content leads to a lower freezing point. This aligns with the general principle that an increase in dissolved solids lowers the freezing point of milk. The freezing point and lactose exhibit a negative correlation, suggesting that lactose contributes to the depression of the freezing point in mare milk.

Milk quality parameters such as protein, lactose, SNF, and TS are closely linked, making them reliable indicators of the overall nutritional value of milk. The negative correlation of the freezing point with SNF and lactose confirms its potential as an indirect measure of milk purity and quality. Although the correlation is weak, the fat content may still contribute modestly to overall density variation due to its lower intrinsic density relative to that of other milk components. The correlations observed suggest that improving one component (e.g., protein) may simultaneously enhance other quality attributes (e.g., SNF, TS, and lactose).

### 3.4. Canonical Discriminant Analysis

#### 3.4.1. Canonical Discriminant Analysis by Lactation Stage

**Figure 3:**
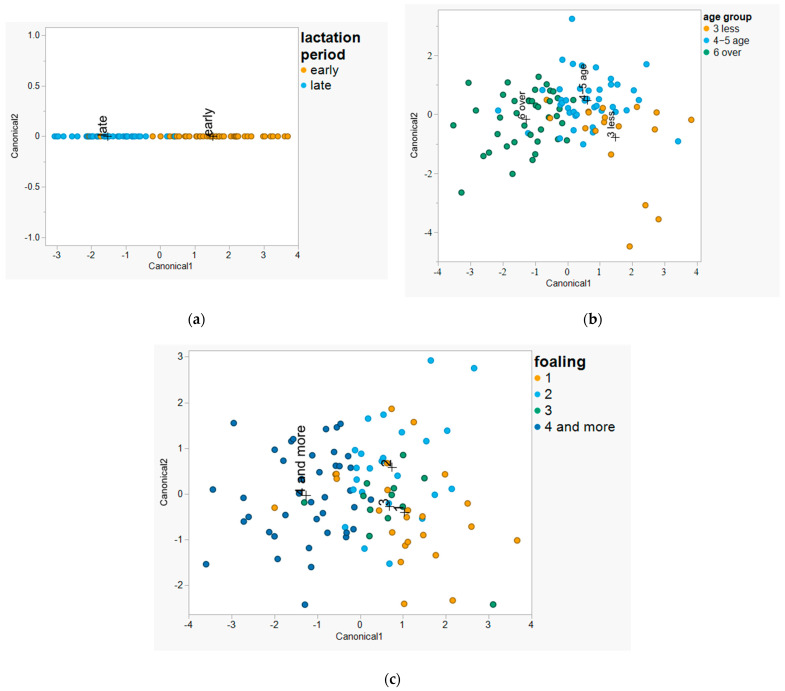
Canonical discriminant analysis scatter plots of milk composition by (**a**) lactation period, (**b**) mare age, and (**c**) foaling number.

The analysis yielded a single canonical function with an eigenvalue of 2.381, accounting for 100% of the discriminant variance. The associated canonical correlation was 0.839, indicating a strong relationship between the discriminant function and group membership. Multivariate significance testing using Wilks’ lambda confirmed that the canonical function significantly discriminated between early- and late-foaling mares (Wilks’ Λ = 0.296, approx. F(14, 85) = 14.457, *p* < 0.0001). This result demonstrates a highly significant difference in milk traits between the early- and late-lactation groups. As shown in the CDA scatter plot (Figure 3), the two groups are clearly separated along Canonical1, with no overlap in Canonical2 (which holds no variance). The standardized canonical coefficients indicated that the most influential variables in separating lactation stages were lactic acid (2.48), the freezing point (0.70), lactose (0.31), and SNF (0.18).

Although the classification plot in Figure 3 shows some proximity between points near the group boundary, the group centroids are completely separated along Canonical1, and no variance is explained by Canonical2. The cross-validation results further support the model’s robustness, with 94% of samples correctly classified by lactation stage.

#### 3.4.2. Canonical Discriminant Analysis by Mare Age

CDA was conducted to assess discrimination based on age (3 years or less, 4–5 years, and 6 years or over). Two canonical functions were extracted and evaluated. The analysis revealed that Canonical Function 1 has an eigenvalue of 1.243, accounting for 84.79% of the total discriminant variance, with a canonical correlation of 0.744, indicating a strong relationship between the discriminant scores and age group membership. Canonical Function 2 contributes an additional 15.21% (eigenvalue = 0.223), with a canonical correlation of 0.427. A multivariate test using Wilks’ lambda confirmed the statistical significance of the overall model (Wilks’ Λ = 0.365, approx. F(28, 168) = 3.936, *p* < 0.0001), indicating that the discriminant functions jointly provide significant separation among age groups. The classification results from the training sample (n = 100) showed an overall correct classification rate of 77.0%, with 23.0% misclassified. The group-specific classification rates are as follows: 3 years or less: 66.7% correctly classified; 4–5 years: 73.8% correctly classified; and 6 years or over: 85.0% correctly classified. Most misclassifications occurred between adjacent age categories, especially within the transitional “4–5 years” group, indicating some overlap in group characteristics. This is consistent with the observed clustering in the CDA scatter plot (Figure 3), where the centroids of the youngest and oldest groups show clearer separation, while the middle group overlaps with both.

#### 3.4.3. Canonical Discriminant Analysis by Foalings Number

In Canonical Function 1, which explains 84.79% of the total variance, the most influential variables are lactic acid (−2.792), acidity (2.176), and total solids (−1.702). This function appears to contrast the acidic profile and solid concentration and is mainly responsible for distinguishing between younger and older mares. In Canonical Function 2, accounting for 15.21% of the variance, acidity (3.481), lactic acid (−3.857), and protein (1.597) are dominant. Only Function 1 shows substantial explanatory power, capturing over 82% of the group separation and demonstrating a strong association between the discriminant scores and foaling number. The multivariate test using Wilks’ lambda indicated that the overall model is statistically significant (Functions 1 to 3: Wilks’ Λ = 0.373, approx. F(42, 246.98) = 2.321, *p* < 0.0001). However, neither Function 2 nor Function 3 is statistically significant on its own (*p* = 0.7554 and *p* = 0.6833, respectively), suggesting that only the first canonical axis meaningfully contributes to group separation. The scatter plot (Figure 3) shows some separation, particularly for mares with four or more foalings, which cluster more to the left on Canonical1. The remaining groups (one, two, and three foalings) are centrally located with substantial overlap, indicating limited discrimination among them. The group centroids confirm this, with the “4 or more” group having the most distinct position.

## 4. Discussion

The present study confirms that lactation stage, mare age, and foaling number significantly affect the chemical composition of Kazakh mare milk. When compared to other breeds commonly used for equine milk production, Kazakh mares demonstrate distinctive traits shaped by centuries of adaptation to extensive pastoral systems. This compositional pattern is not unique to Kazakh horses. Similar traits have been reported in both native and crossbred horse breeds raised under semi-arid and mountainous conditions in regions such as Siberia, Mongolia, and Eastern Europe. For instance, historical and recent data compiled by the FAO confirm that regional breeds show adaptive lactation profiles that reflect long-standing selection for resilience and productive performance under extensive, pasture-based systems. These parallels suggest that Kazakh mares share a broader pattern of ecological and genetic adaptation among Eurasian horse breeds traditionally used for milk production [26].

The protein content in the Kazakh mare milk was observed to increase in late lactation (1.96%) compared to in early lactation (1.82%) (*p* = 0.0003), which aligns with the findings in thoroughbred and Haflinger mare milk, where the protein content was also found to increase during the mid-to-late lactation stages (up to ~2.0%) due to the natural transition from energy-focused to structure-supportive milk composition [1,2,29]. The protein content in Arabian mare milk varies during lactation. According to Pieszka (2005) [30], the total protein concentration was the highest at the beginning of lactation at 3.43%, and then, it decreased to 2.09% within a month [30]. Another study reported that the average protein content in Arabian mare milk was 2.13% [31]. Age-related changes also influence casein synthesis, with the milk produced by mares aged 4–7 years typically having a higher total casein content than that produced by very young or older animals, likely reflecting optimal glandular function and hormonal balance [25]. This trend was confirmed in our results, where casein concentrations were significantly higher in the milk from mares older than four years (1.38–1.39%) than in the milk from younger mares (1.31%), indicating a statistically significant effect of age on casein yield.

However, Kazakh mare milk generally presents a lower fat content (0.40% early; 0.35% late) than Haflinger (~1.0–1.2%) or Arabian mare milk (~0.7–1.1%) [3]. Haflinger mares produce milk with a fat content of approximately 1.69%, with the saturated-to-unsaturated fatty acid ratio ranging from 1.09% to 1.29% throughout the six months of lactation [32]. In contrast, the Lipizzaner breed produces milk with an even lower mean fat content of about 1.18%, though the fat content in individual samples ranges from as low as 0.25% to as high as 3.04% during lactation. These wide fluctuations may be attributed to individual metabolic differences or environmental factors affecting pasture quality [33]. Milk samples from Polish Konik and Wielkopolski breeds show a mean fat content of 1.21% [34]. This lower fat concentration may reflect breed-specific metabolic adaptations and the influence of extensive grazing conditions in the Kazakh steppe.

The content of lactose, a major energy source for foals, was significantly higher in the late-lactation milk (7.10%) than in the early-lactation milk (6.74%) (*p* = 0.0001) from Kazakh mares. This is slightly above the values reported in milk from other breeds, where lactose typically ranges from 6.0 to 6.8% [35]. Higher values are typically reported in thoroughbred (6.0–6.5%) and Haflinger mare milk (~6.4–6.8%) [1,22]. In Arabian mare milk, lactose levels typically range between 6.2% and 6.6% during early lactation, with minor variations over time [30]. Supplementary feeding, particularly with energy-rich components, is associated with a significant increase in milk lactose concentrations and improved metabolic support for lactose synthesis in lactating mares [36]. This is a beneficial feature, as a high lactose content contributes to the characteristic sweet taste of kumis and supports effective fermentation during traditional dairy processing. The elevated lactose levels may reflect the mares’ adaptation to forage-rich pastures during late spring and summer, enhancing carbohydrate synthesis in their milk.

The SNF and TS were also significantly higher in the late-lactation milk (9.89% and 10.36%, respectively), indicating a general enhancement of nutritional density over time. The TS and SNF contents vary among breeds. For instance, Lipizzaner mare milk demonstrates TS values ranging from 9.5% to 10.5%, influenced by lactation stage and feeding practices [37]. These values fall within or slightly above the range reported for other breeds, where SNF ranges from 8.5 to 9.8%, and TS ranges from 9.5 to 11% [26], suggesting that Kazakh mares are capable of producing competitively nutrient-dense milk even under semi-extensive conditions. The SNF and TS levels in milk from Kazakh mares are comparable or slightly superior to those in milk from other native breeds, such as the Altai or Yakut [14], which are better known for producing milk with high fat but moderate SNF levels. During early lactation, the milk density tends to be higher due to elevated protein levels in the colostrum. As lactation progresses, the increase in protein and lactose contents contributes to an increase in milk density, resulting in more stable physicochemical properties in mature milk [38].

The urea and acidity levels in Kazakh mare milk increased with mare age and foaling number. These trends have been reported in Haflinger and Italian saddle breeds, where prolonged lactation and repeated reproductive cycles are associated with metabolic shifts resulting in elevated nitrogen byproducts and increased milk acidity [23]. The acidity of mare milk is the highest in the immediate post-foaling colostrum, contributing to antimicrobial protection and gut microbiota establishment in the foal, while it gradually decreases during lactation as the milk transitions to a more stable composition with a higher lactose content and a lower protein concentration [36]. However, the high lactose and moderate protein profile of milk from Kazakh mares, particularly that from younger individuals, makes it more similar to human milk, which is often cited as having lower protein and fat contents than ruminant milk. Lactic acid, which is produced through natural fermentation processes, contributes to the acidity of mare milk, especially under fermentation conditions [39]. For instance, Arabian mare milk exhibits a lower acidification rate during lactic acid fermentation than cow milk, resulting in a different coagulum structure [40].

The freezing point of mare milk serves as an important physicochemical parameter, reflecting the concentrations of soluble constituents such as lactose, salts, and minerals. It is considered a sensitive indicator of milk authenticity and composition, and it shows variation across breeds and lactation stages. Notably, the freezing point depression observed in milk from early lactation and from mares with fewer foalings suggests a higher concentration of dissolved solids, which has also been reported in thoroughbred mare milk obtained during colostral and early-lactation phases [24]. In Polish coldblood mare milk, the freezing point was reported on average to be −0.550 °C during mid-lactation, showing remarkable stability and suggesting a consistent osmotic composition during this lactation period [41]. Similarly, in Croatian coldblood mare milk, the freezing point ranged from −0.5423 °C in the first month to −0.5221 °C in the second month of lactation, reflecting early lactational changes in the concentration of solutes such as lactose [42]. Breed-specific differences have also been observed in Italian saddle and Haflinger mares. The freezing point of Italian saddle mare milk ranged from −0.529 to −0.533 °C, while that of Haflinger mare milk varied from −0.531 to −0.522 °C [23]. Our results showed significant differences in the freezing point parameter according to foaling number, lactation stage, and age. These slight but consistent variations likely arise from differences in the citric acid content, given its strong influence on the milk’s freezing point depression.

In summary, while the milk of Kazakh mares shares many compositional traits with that of other breeds—such as increasing protein and TS contents during lactation—it stands out for its low fat, high lactose, and moderate protein contents, especially under natural pasture-based systems. This makes Kazakh mare milk particularly suitable for kumis fermentation and potential functional food applications, especially for populations seeking milk closer in composition to human milk. Moreover, the breed’s adaptation to pasture conditions reinforces its value in sustainable equine dairy systems.

## 5. Conclusions

The results of this study confirm that the chemical composition of milk obtained from mares of the Kazakh breed is significantly influenced by lactation stage, mare age, and foaling number. A key aspect of the experiment was that all mares were kept exclusively on natural pastures, without any supplementary feeding, allowing for an objective assessment of their inherent productive potential.

Kazakh mare milk exhibited consistently high values in terms of lactose content, density, total solids, protein, and casein. Particularly notable were the lactose and protein levels, which exceeded those typically reported in milk from other equine breeds. This makes Kazakh mare milk suitable not only for direct consumption but also for traditional fermentation products such as kumis.

These findings highlight the value of Kazakh horses as a unique genetic resource for sustainable equine husbandry. Due to the absence of concentrated feeds or chemically treated fodder, their milk can be considered environmentally friendly and qualifies as organic. This presents promising opportunities for positioning mare milk as a premium, high-quality product that aligns with current trends in sustainable agriculture and healthy nutrition.

Therefore, Kazakh mare milk has strong potential in the organic food market, particularly in niche sectors focused on traditional diets, natural functional beverages, and therapeutic or preventative nutrition. As a logical continuation of the current research, the next phase of work with Kazakh dairy mares as a source of organic milk will focus on identifying biologically active compounds, including peptides, amino acids, antioxidants, and immunomodulators, as well as evaluating their potential pharmacological properties. Additionally, a comparative analysis will be conducted to assess the presence of these compounds in the milk of Kazakh mares kept exclusively on natural pastures without supplementary feeding compared to mares housed in stables with additional feeding, including concentrated feeds.

## Figures and Tables

**Table 1:** Chemical composition of milk obtained from Kazakh mares according to lactation period, mare age, and foaling number.

	Lactation Period		Mare Age			Foaling Number				*p*-Value		
Parameter	Early (*n* = 50)	Late (*n* = 50)	3 Years (*n* = 18)	4–5 Years (*n* = 42)	6 Years Over (*n* = 40)	1 (*n* = 24)	2 (*n* = 24)	3 (*n* = 12)	4 or More (*n* = 40)	Lactation	Age	Foaling
Fat (%)	0.402 ± 0.031	0.351 ± 0.024	0.421 ± 0.044 ^a^	0.394 ± 0.032 ^ab^	0.351 ± 0.022 ^b^	0.441 ± 0.038 ^a^	0.418 ± 0.037 ^a^	0.300 ± 0.052 ^b^	0.348 ± 0.022 ^b^	0.2370	0.0194	0.0129
Protein (%)	1.819 ± 0.032 ^b^	1.958 ± 0.091 ^a^	1.821 ± 0.051	1.917 ± 0.021	1.901 ± 0.020	1.849 ± 0.043	1.944 ± 0.034	1.860 ± 0.062	1.901 ± 0.016	0.0003	0.0667	0.0702
SNF (%)	9.354 ± 0.487 ^b^	9.888 ± 0.206 ^a^	9.542 ± 0.417	9.682 ± 0.410	9.606 ± 0.520	9.532 ± 0.429	9.711 ± 0.393	9.718 ± 0.393	9.61 ± 0.52	0.0001	0.4560	0.4607
TS (%)	9.813 ± 0.623 ^b^	10.363 ± 0.322 ^a^	10.060 ± 0.683	10.163 ± 0.473	10.049 ± 0.590	10.061 ± 0.661	10.189 ± 0.504	10.062 ± 0.334	10.049 ± 0.590	0.0001	0.3627	0.3102
Lactose (%)	6.741 ± 0.307 ^b^	7.073 ± 0.202 ^a^	6.908 ± 0.318	6.950 ± 0.238	6.905 ± 0.344	6.930 ± 0.291	6.914 ± 0.252	7.001 ± 0.241	6.906 ± 0.344	0.0001	0.2572	0.2522
Galactose (%)	0.328 ± 0.061	0.347 ± 0.051	0.338 ± 0.049 ^a^	0.328 ± 0.050 ^b^	0.344 ± 0.063 ^a^	0.328 ± 0.063 ^b^	0.340 ± 0.039 ^b^	0.343 ± 0.023 ^b^	0.348 ± 0.063 ^a^	0.1984	0.0438	0.0302
Glucose (%)	0.247 ± 0.070	0.268 ± 0.043	0.266 ± 0.064 ^a^	0.243 ± 0.053 ^b^	0.280 ± 0.043 ^a^	0.253 ± 0.062 ^b^	0.240 ± 0.041 ^b^	0.227 ± 0.062 ^b^	0.279 ± 0.043 ^a^	0.0918	0.0038	0.0111
Freezing point (°C)	−0.528 ± 0.034 ^a^	−0.588 ± 0.014 ^b^	−0.559 ± 0.023 ^b^	−0.570 ± 0.043 ^a^	−0.549 ± 0.032 ^b^	−0.561 ± 0.024 ^b^	−0.569 ± 0.033 ^a^	−0.583 ± 0.034 ^a^	−0.564 ± 0.032 ^b^	0.0001	0.0042	0.0023
Acidity °Dornic (°D)	8.422 ± 0.184	8.231 ± 0.109	8.490 ± 0.291 ^b^	8.594 ± 0.161 ^b^	7.978 ± 0.142 ^a^	8.524 ± 0.261 ^b^	8.647 ± 0.220 ^b^	8.474 ± 0.232 ^b^	7.978 ± 0.142 ^a^	0.3629	0.0088	0.0152
Lactic acid (%)	0.104 ± 0.001	0.102 ± 0.007	0.105 ± 0.002 ^b^	0.106 ± 0.001 ^b^	0.099 ± 0.001 ^a^	0.105 ± 0.002 ^b^	0.106 ± 0.002 ^b^	0.104 ± 0.002 ^b^	0.099 ± 0.001 ^a^	0.4099	0.0071	0.0126
Density (g/L)	1039.822 ± 3.592 ^b^	1043.191 ± 1.021 ^a^	1041.522 ± 3.093 ^b^	1042.438 ± 1.990 ^b^	1040.520 ± 3.801 ^a^	1041.825 ± 2.801 ^b^	1042.395 ± 2.011 ^b^	1042.343 ± 2.279 ^b^	1040.520 ± 3.801 ^a^	0.0001	0.0052	0.0196
Citric acid (%)	0.150 ± 0.024	0.149 ± 0.013	0.147 ± 0.012	0.141 ± 0.020	0.149 ± 0.022	0.142 ± 0.009	0.153 ± 0.019	0.149 ± 0.011	0.143 ± 0.022 ^a^	0.1686	0.4583	0.0456
Urea (mg/dL)	27.834 ± 1.034 ^a^	24.674 ± 0.690 ^b^	27.886 ± 0.932 ^b^	28.151 ± 1.141 ^b^	23.523 ± 0.810 ^a^	27.214 ± 1.474 ^b^	28.639 ± 1.331 ^b^	28.658 ± 1.554 ^b^	23.523 ± 0.810 ^a^	0.0127	0.0008	0.0015
Casein (%)	1.340 ± 0.024 ^b^	1.398 ± 0.081 ^a^	1.312 ± 0.042 ^b^	1.378 ± 0.020 ^a^	1.388 ± 0.007 ^a^	1.334 ± 0.035	1.384 ± 0.022	1.351 ± 0.048	1.386 ± 0.012	0.0342	0.0456	0.1457

Values are presented as the mean ± SD. Superscript letters (a, b) indicate significant differences within rows, determined using Tukey’s HSD test (*p* < 0.05). Only parameters with statistically significant differences are labeled. SNF, solids-not-fat; TS, total solids.

## Data Availability

The data are contained within the article.

## Abbreviations

The following abbreviations are used in this manuscript:

SNF	solids-not-fat
TS	total solids
PCA	principal component analysis
CDA	canonical discriminant analysis
ANOVA	analysis of variance
PC	principal component
